# Supplementary material for: Adipokines and Metabolic Regulators in Human and Experimental Pulmonary Arterial Hypertension
Source: Int J Mol Sci. 2021 Feb 1;22(3):1435. doi: 10.3390/ijms22031435 (PMC7867052; doi:10.3390/ijms22031435)
Supplement: Supplementary file 1 [file ijms-22-01435-s001.pdf]

Figure S1. Experimental design

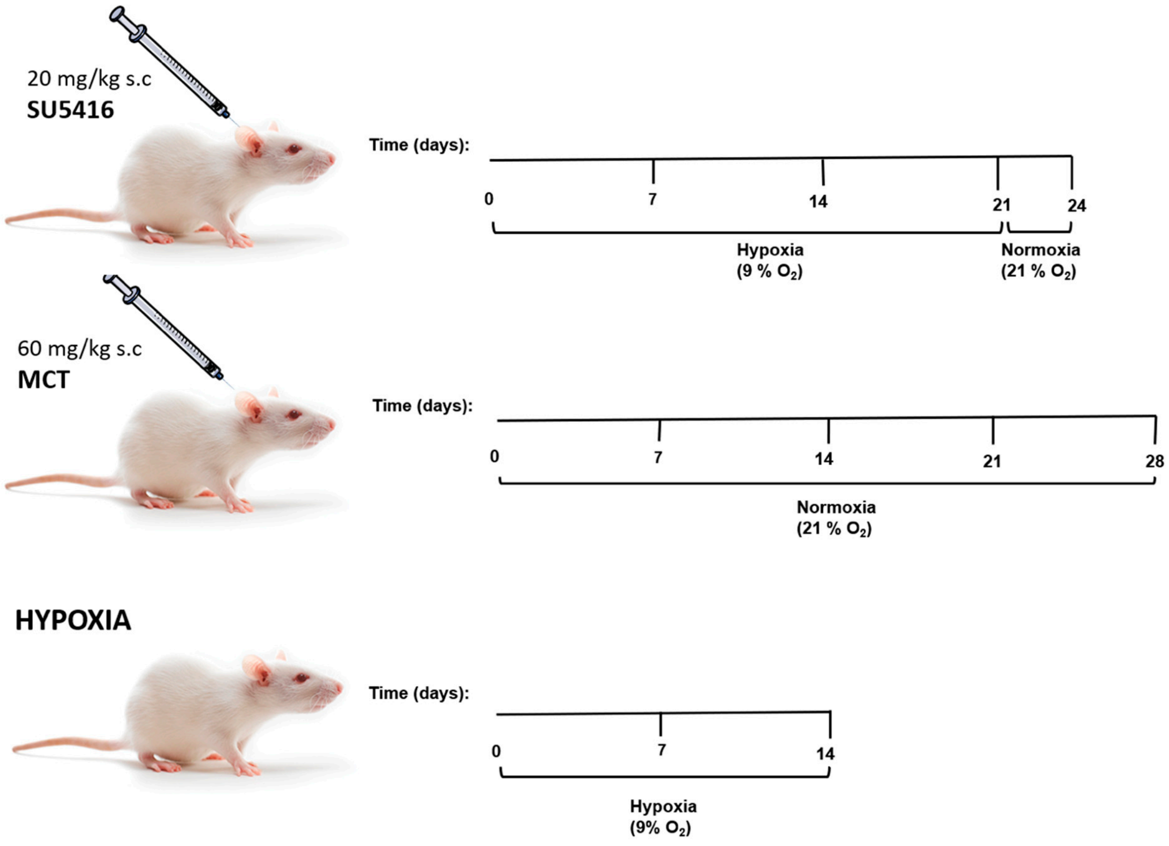

Table S1. Demographics and clinical information of human patients with IPAH and controls						
	Human serum cohort			Human lung tissue cohort		
	**IPAH**<br>**N = 15**	**Controls**<br>**N = 22**	**P value**	**IPAH**<br>**N = 12**	**Controls**<br>**(Failed donors)**<br>**N = 12**	**P value**
**Demographic and Somatometric Characteristics**						
Age [mean ± SD]	38.4 ± 18.6	48.3± 10.9	0.128	38.5 ± 13.4	39.9± 14.5	0.805
Gender (M/F)	4/11	4/18	0.142	6/6	6/6	1
Race (White)	14	22	0.220	12 (100)	12 (100)	1
BMI [mean ± SD], Kg/m^2	24.9 ± 8.4	24.6 ±3.8	0.819	NA	NA	-
**Medical history - Comorbid Conditions at Enrollment**						
History of COPD	0	0	-	1 (8.3)	0	0.307
Heart Failure	5 (33.3)	0	0.004	5 (41.7)	0	0.012
ICD	1 (6.7)	0	0.220	0	0	-
Ischemic Stroke	0	0	-	0	0	-
Arterial Hypertension	4 (26.7)	0	0.010	2 (16.7)	4 (33.3)	0.346
Type 1 diabetes mellitus	1 (6.7)	0	0.220	0	1	0.307
Cancer	1 (6.7)	0	0.220	0	0	-

Thyroid disorders	3 (20)	0	0.053	4 (33.3)	0	0.028
Rheumatoid arthritis	0	0	-	0	0	-
Smokers	3 (20)	NA	-	5 (41.7)	8 (66.7)	0.219
Recreational drug user	0	NA	-	1 (8.3)	6 (50)	0.025
**PAH- specific medications**						
Endothelin Receptor antagonist (ETA)	5 (33.3)			1 (8.3)		
Dual Endothelin Receptor antagonist (ETA+ETB)	4 (26.7)			2 (16.7)		
Phosphodiesterase (PDE) inhibitors	8 (53.3)			5 (41.7)		
Prostacyclin analogues	7 (46.7)			5 (41.7)		
Calcineurin inhibitor	1 (6.7)			0		
**Current Concomitant medications**						
Anticoagulant (%)	12			7 (58.3)		
Antiplatelet	4 (26.7)			3 (25)		
Calcium Channel Blocker	1 (6.7)			0		
Diuretic	11 (73.3)			11 (91.7)		
Digoxin	11 (73.3)			7 (58.3)		
Psychotropic drugs	3 (20)			4 (33.3)		
SSRI	1 (6.7)			1 (8.3)		
Synthetic Thyroid Replacement	3 (20)			3 (25)		

Table S2. Functional class, hemodynamics and laboratory data of IPAH patients

Abbreviations: BMI, body mass index; **SD**, standard deviation**; COPD**, chronic obstructive pulmonary disease; **ICD**, Ischemic Cardiovascular Disease (Previous MI, History of CVA, PAD Angina pectoris) **NA,** not applicable;

Data are described as mean (SD) for continuous variables and counts (%) for categorical values.

	**Human serum cohort Patients (N= 15)**	**Lung tissue cohort Patients (N=12)**
WHO functional class I	0	0
WHO functional class II	2 (13.3)	0
WHO functional class III	9 (60)	4 (33.3)
WHO functional class IV	4 (26.7)	6 (50)
6MWT [mean ± SD], (m)	332.2 (123)	292 (137)
**Pulmonary Function Testing**		
FEV1/FVC ratio	74 (8.1)	71.5 (6.4)
Single breath DLCO (mL/min/mmHg)	15.9 (5.9)	20.9 (5.7)
Percent predicted DLCO (%)	70 (21.5)	72 (29.5)
**Hemodynamic parameters**		
mPAP (mmHg)	62 (24.5)	58.5 (14.2)
CI (L/min/m^2)	2.06 (1.53)	2.02 (0.76)
PVR mean, Woods units	13.4 (6.7)	11.7 (8.9)
PCWP (mmHg)	12 (3.2)	12.5 (5.0)
DBP (mmHg)	60 (10)	60 (4.2)
SBP (mmHg)	100 (19)	101 (19.5)
No significant MV, AV or LH disease (Doppler Echocardiogram)	15 (100)	12 (100)

Laboratory blood test		
Hematocrit (%)	35 (11.6)	36 (12)
White Blood Cells (10^9/L)	6.740 (5.000)	5.750 (3.130)
Platelets (10^9/L)	155 (64)	133 (72)
Creatinine (mg/dl)	0.90 (0.25)	1.01 (0.32)
Alkaline phosphatase (U/L)	69.5 (24.9)	105 (55)
Aspartate transaminase (IU/L)	23 (14.5)	18 (5)
Alanine transaminase (IU/L)	19 (16)	25 (13)
Total bilirubin (mg/dl)	0.80 (0.38)	0.60 (0.60)
Uric Acid (mg/dl)	7.7 (2.0)	

Abbreviations: IPAH, Idiopathic pulmonary arterial hypertension; **SD**, standard deviation**; 6MWT**, 6-minute walking test; **WHO,** world health organization; **MV**, mitral valve; **AV**, aortic valve; **LH**, left heart; **DBP**, Diastolic blood pressure; **SBP**, Systolic blood pressure; **FEV1**, forced expiratory volume in 1 second **; FVC**, forced vital capacity; **DLCO**, Diffusion Lung Capacity Oxygenation; **mPAP**, mean pulmonary arterial pressure; **CI,** cardiac index; **PVR**, pulmonary vascular resistance; **PCWP**, pulmonary capillary wedge pressure; PVR and CI calculated by Fick method. Data are described as median (IQR) for continuous variables and counts (%) for categorical values.

Table S3. Primer sequences used for quantitative polymerase chain reaction				
**Gene**	**Species**	**Accession No**	**Forward**	**Reverse**
18S*	Rat<br>Human	M11188<br>NR_146146.1	GCAATTATTCCCCA<br>TGAACG	GGCCTCACTAAACCA<br>TCCAA
FABP-4	Rat	NM_053365	AGTGAAGAGCATCA<br>TAACCC	TGTAGAAGTCACGCC<br>TTTC
PPARγ	Rat	NM_013124.3	GTCTCACAATGCCA<br>TCAGG	AGCAGACTCTGGGTT<br>CAG
ACAD-S	Rat	NM_022512.2	GGGCCTCATCTACA<br>GCTAA	CAGGGTTTGCATGGC<br>TATC
ACAD-M	Rat	NM_016986	GGGTTTAGCTTCGA<br>GTTGAC	TGAGAGGGAACGGGT<br>ATTC
ACAD-VL	Rat	NM_012891.2	CTAGGAGAAGTGGG<br>AGATGG	CATGATCAACCGCCT<br>TGG
CD36	Rat	NM_031561.2	CATGCAAGTCCTGA<br>TGTCTC	CAGTTATGGGTTCCA<br>CATCC
GLUT1	Rat	NM_138827.1	TTCGGCTTAGACTC<br>CATCA	GAAGGGCAACAGGAT<br>ACAC
GLUT4	Rat	NM_012751.1	CTCAATGGTTGGGA<br>AGGAAA	CCGTCCGAGAATGAG<br>TATCT
PFK-1	Rat	NM_031715.1	CCAATCTGTGTGTC<br>ATCGG	GCTGTGATCTTCCCAT<br>CTTT
PDH-b	Rat	NM_0010076<br>20	GACAGTTCGTGAAG<br>CCATTA	GCCTCTGCTAACCTT<br>GTATG

CS	Rat	NM_130755.1	GCCAGAAACTGCTACCTAAG	AAGAGACCTGTTCCTCTGT
Adiponectin receptor 1	Rat	NM_207587.1	TCCTGACTGGCTGAAAGA	GTGTGGATGCGGAAGATG
Adiponectin receptor 2	Rat	NM_001037979.1	CACGATGACAACTCCCAAG	ACCTTCCCACACCTTACA
Adiponectin	Rat	NM_144744	GTGCCAGTGGATCTGATTAC	CATGACTGGGCAGGATTAAG
FGF21	Rat	NM_130752.1	CTATGGATCGCCTCACTTTG	ATCCTGGGAGTCCTTCTG
PPAR-α	Rat	NM_013196.1	GATTTCTCAGTCCCTCGGA	AGAGAGGGTGTCTGTGATG
PDK-4	Rat	NM_053551.1	AGAGCTGGTACATCCAGAG	GACCAGCGTGTCTACAAAG
ACC-2	Rat	XM_017598246.1	ACAGTCCAAGAAACACATCC	CTCCAAGTGGCGGTAAATC
FGF21	Human	NM_019113.3	GCCAGAGGCTGTTTACTATG	ATCCTCCTCTGGAACTCTTT
FABP4	Human	NM_001442.2	GCA TGG CCA AAC CTA ACA	GGC CCA GTA TGA AGG AAA TC
PPARγ	Human	NM_138712.3	CGAGAAGGAGAAGCTGTTG	CAGCGGGAAGGACTTTATG

Definition of genes: FABP-4: Fatty acid binding protein-4, PPARγ: Peroxisome proliferator-activated receptor gamma, ACAD-S: short-chain acyl-coenzyme A dehydrogenase, ACAD-M: medium-chain acyl-coenzyme A dehydrogenase, ACAD-VL: very long-chain acyl-coenzyme A dehydrogenase

CD36: cluster of differentiation 36, GLUT-1: Glucose transporter-1, GLUT-4: Glucose transporter-4, PFK-1: Phosphofructokinase-1, PDH-b: Pyruvate Dehydrogenase-beta subunit, CS: Citrate synthase, FGF-21: Fibroblast growth factor-21. PPARα: Peroxisome proliferator-activated receptor alpha, PDK-4: Pyruvate Dehydrogenase Kinase 4, ACC-2: Acetyl-CoA carboxylase-2
